# Association of Context-Specific Sitting Time with Healthcare Costs at Midlife: The Northern Finland Birth Cohort 1966 Study

**DOI:** 10.1249/MSS.0000000000003995

**Published:** 2026-03-26

**Authors:** SANAZ AKHAVAN RAD, ANNA-MAIJU LEINONEN, RAIJA KORPELAINEN, MIKKO VAARAMO, FRANK KIWANUKA, KATJA RYYNÄNEN, KRISTIINA PATJA, PAULUS TORKKI

**Affiliations:** 1 Department of Public Health, Faculty of Medicine, University of Helsinki, Helsinki, FINLAND; 2 Research Unit of Population Health, Faculty of Medicine, University of Oulu, Oulu, FINLAND; 3 Department of Sports and Exercise Medicine, Oulu Deaconess Institute Foundation sr, Oulu, FINLAND; 4 Research Unit of Health Sciences and Technology, Faculty of Medicine, University of Oulu, Oulu, FINLAND; 5 Medical Research Center Oulu, Oulu University Hospital, University of Oulu, Oulu, FINLAND; 6 Department of Economics, Turku School of Economics, University of Turku, Turku, FINLAND; 7 Department of Nursing Science, Faculty of Health Sciences, University of Eastern Finland, Kuopio, FINLAND; 8 School of Graduate Studies, Research and Innovations, Clarke International University, Kampala, UGANDA

**Keywords:** COHORT STUDY, ECONOMIC BURDEN, LEISURE BEHAVIOR, OCCUPATIONAL HEALTH, PUBLIC HEALTH, SEDENTARY BEHAVIOR

## Abstract

**Purpose:**

Prolonged sitting is linked to poor health outcomes; however, its context, particularly work versus leisure, and its economic impact are less understood. This study examined the extent and context of daily sitting time and its association with annual healthcare costs at midlife in a large Finnish cohort.

**Methods:**

A sample (*n* = 7147) of Finnish adults at the 46-yr follow-up point of the Northern Finland Birth Cohort 1966 study was used. Data were collected between 2012 and 2014, including self-reported sitting time (work and leisure) and healthcare costs estimated from self-reported visits to public, private, and occupational primary healthcare services using unit costs based on the Finnish Institute for Health and Welfare. Finally, a generalized linear model (gamma distribution with log link), was used to examine the associations between sitting time and annual healthcare costs.

**Results:**

Total sitting time was 7.3 ± 3.3 h·d^−1^, including 4.0 ± 2.2 h·d^−1^ during leisure and 3.4 ± 2.6 h·d^−1^ at work. In adjusted generalized linear model analyses, participants in the second quartile of work-related sitting (1.01–3.00 h·d^−1^) had approximately 20% higher annual primary healthcare costs compared with those in the lowest quartile (*P* = 0.025). Leisure-time sitting showed no independent association.

**Conclusions:**

In this population-based cohort, work-related sitting, but not leisure-time sitting, was associated with higher annual primary healthcare costs. These findings highlight the potential economic impact of occupational sedentary behavior and suggest that work-related sitting should be considered in future strategies and studies addressing sedentary behavior in workplace settings.

Sedentary behavior (SB) is a public health concern, particularly in high-income countries (1); SB is defined as any waking activity in a sitting, reclining, or lying posture with ≤1.5 metabolic equivalents of energy expenditure (2). In Finland, adults are sedentary for almost 60% of their waking hours, mostly while sitting, which adds up to over 8 h·d^−1^ (3). Prolonged sitting refers to extended daily sitting time (e.g., ≥8–12 h·d^−1^) accumulated across waking hours (4). Prolonged sitting is linked to poor health outcomes such as increased risks of type 2 diabetes, heart disease, and cancer (5,6), which persist even among individuals who exercise regularly (7,8).

Prolonged sitting leads to physiological changes that affect cardiometabolic health (9). Studies show that uninterrupted sitting decreases muscle contractions (10) and blood flow, leading to lower glucose uptake, reduced lipoprotein lipase activity, and impaired endothelial function (11). These mechanisms contribute to insulin resistance and dyslipidemia (12), helping to explain why higher sedentary time is linked to greater risk of chronic disease and increased healthcare needs.

Beyond health effects, sitting for long periods also contributes to economic costs; for instance, sitting for 8 or more hours per day was linked to an estimated €1.5 billion in healthcare costs in Finland in 2017 (13). Similarly, sedentary time accounted for approximately £0.7 billion annually in National Health Service costs in the United Kingdom, and excessive SB (>8 h·d^−1^) accounted for about CAD $2.2 billion or 1.6% of total illness costs in Canada (14,15). Even so, some studies have reported no clear link between sitting time and healthcare expenditure after considering factors such as physical activity or chronic illness (16,17). However, previous studies have only focused on total sedentary time when examining associations with healthcare costs. This approach does not account for different sitting contexts, such as time spent sitting at work versus during leisure (2,18). As a result, it remains unclear whether the economic burden of sitting arises from occupational constraints, discretionary leisure behaviors, or both, limiting the development of targeted prevention and cost-reduction strategies.

Recent evidence suggests that prolonged sitting contributes to nearly 60% of the total daily sitting time among desk-based workers, pointing to the workplace as a key target for interventions aiming to reduce sitting time (19). Conclusively, a systematic review and meta-analysis have shown stronger links between work-related sitting and back or neck pain compared with sitting outside the work domain (20). Given that SB is shaped by different factors in the work and leisure settings, this underscores the potential variation in sitting time across these contexts (21). Thus, it is important to consider where and why sitting occurs.

Although one study did not directly assess sitting by domain, it emphasized the importance of considering context-specific SBs, such as TV viewing, occupational sitting, and transport-related sitting, as each may have different causes and require specific types of intervention (22), thus illuminating the importance of examining the extent and context of daily sitting time (e.g., work versus leisure sitting).

Evidence shows that work and leisure sitting relate differently to health and behavioral patterns (23), which further indicates that domain-specific sitting provides information that total sedentary time alone cannot capture. Therefore, because work and leisure sitting differ in behavioral contexts (24), they may have distinct associations with healthcare costs and require different intervention approaches, particularly in occupational and policy settings. Occupational sitting, which often occurs in settings where movement is restricted and workers have limited opportunities to interrupt prolonged sitting, has been linked to altered hemodynamic regulation and autonomic stress responses during working hours (25), as well as higher long-term risks of all-cause and cardiovascular mortality (26). In contrast, leisure-time sitting often co-occurs with passive screen use, low energy expenditure, and other unhealthy behaviors, contributing more directly to adverse metabolic outcomes and reduced psychological well-being (27). Together, these findings suggest that the health and cost implications of sitting depend not only on total sedentary time but also on the domain in which it occurs.

The patterns of prolonged sitting are also shaped by broader societal and environmental factors, including education level, occupation, and income (28). In line, evidence has revealed that occupational groups may influence the extent of SB across occupations (29). For instance, occupations within the transport industry, particularly truck driving, exemplify a high-risk occupational group where extended sitting is compounded by other stressors such as isolation, limited opportunities for physical activity, and irregular schedules (30). These combined risk factors have been associated with higher rates of work-related injuries, chronic illnesses, and psychological distress, contributing to increased healthcare costs (31)

Although current guidelines highlight the significance of SB and physical activity (32), current evidence links physical inactivity to higher healthcare costs (33). The specific association between sitting time—a distinct domain of SB—and healthcare costs is still not widely studied, highlighting the need for further investigation.

To address these gaps, this study examined the associations between domain-specific sitting time, that is, during work and leisure, and annual healthcare costs at midlife (at the 46-yr point) in a large population-based Finnish birth cohort. In addition, variations in total sitting duration across occupational groups were also assessed. We hypothesized that higher work-related and leisure sitting time is associated with higher annual primary healthcare costs. We also hypothesized that there would be differences in their impact on healthcare costs.

## METHODS

### Data Source and Participants

This study utilized data from the Northern Finland Birth Cohort 1966 (NFBC1966) study, a population-based longitudinal study established to examine biological, behavioral, and socioeconomic determinants of health across the life course (34). The cohort originally included 12,231 children born to expectant mothers residing in the northernmost provinces of Finland (Oulu and Lapland) in 1966, covering approximately 13.2% (604,000 inhabitants) of the national population and 48% (160,000 km^2^) of the Finnish land area at the time.

The current analysis used data from the 46-year follow-up (2012–2014) of the NFBC 1966 study. This time point was selected because it was the first follow-up to include detailed questions on sitting behavior across multiple domains (work, home, and transport) and self-reported primary healthcare use, enabling analysis of their interrelationship. Age 46 represents midlife, when SB patterns are typically stable and healthcare utilization begins to increase, making it a relevant stage for examining the economic implications of sitting.

During this follow-up, 7146 participants (69.2% of the original cohort) completed postal questionnaires (35). Because healthcare cost data were available only for participants who answered the survey questions regarding their use of health services, the subanalysis on healthcare costs and sitting time only included 4334 participants. We intentionally chose to include descriptive statistics based on the entire sample (*n* = 7147) to provide a comprehensive overview of participants who participated in the 46-yr follow-up survey.

Ethical approval was obtained from the Regional Ethics Committee of the Northern Ostrobothnia Hospital District (EETTMK 94/11). All participants provided written informed consent in accordance with the Declaration of Helsinki.

### Exposure and Outcome Measures

The primary exposure was self-reported weekday sitting time, measured during the 46-yr follow-up. Participants were asked the following question: “How many hours do you sit on average on a weekday?,” with sitting reported separately across five domains: 1) during the working day in an office or similar environment, 2) at home watching movies or television, 3) at home using a computer, and 4) in vehicles, and 5) elsewhere.

For the present analysis, sitting was classified into work-related and leisure-time domains. Work-related sitting was defined as time reported “at work or similar.” Leisure-time sitting was calculated as the sum of time spent “watching TV or videos,” using a computer at home,’ and “sitting in a vehicle.” Sitting in a vehicle was included in the leisure-time domain because it represented a relatively small proportion of total daily sitting time and combining it with leisure sitting reduced low-frequency exposure categories and improved the stability of regression estimates. Sitting reported as “elsewhere” was excluded because the context was not sufficiently defined. Since data were collected in 2012, when remote or hybrid work was uncommon in this cohort, home computer use was treated as leisure-time sitting.

Responses were recorded in hours, and values were summed and reported as the mean total sitting time in hours per day across domains of work and leisure settings (36). Following a previously established approach, participants reporting sitting times exceeding 18 h·d^−1^ (*n* = 27) were excluded from the analysis to reduce implausible reporting. This cutoff is consistent with prior NFBC 1966 analyses—skewed values (e.g., 18 h of sitting or higher represent implausible values likely due to reporting error rather than true behavior (37)).

Occupational data were collected using the question: “What is your current profession or occupation?” In this study, responses were linked to occupational codes based on Statistics Finland’s Classification of Occupations (adapted from the International Standard Classification of Occupations) and subsequently classified based on physical demand into four groups: 1) office-based work (mostly involving sitting, e.g., directors, senior management, advisors, officials, office workers, and customer service representatives), 2) sitting and walking work (e.g., process and transport workers), 3) walking and lifting (e.g., service, sales, care staff, and soldiers), and 4) strenuous labor (e.g., farmers, construction, repair, and manufacturing workers). Mixed-task occupations were classified according to the dominant physical demand based on their primary International Standard Classification of Occupations code. This occupational categorization aligns with Statistics Finland’s physical-demand–based classification and previous Finnish studies (38,39).

The primary outcome was the direct primary healthcare cost at the 46-yr follow-up point. In Finland, primary healthcare includes public healthcare offered by municipalities, occupational clinics, and private clinics. Costs were identified based on self-reported data on all primary healthcare visits during the previous year. Cost was calculated by multiplying the number of self-reported visits with the unit costs of each visit provided by the Finnish Institute for Health and Welfare (THL) Unit Costs of Health and Social Care report (40). This report supports national health expenditure monitoring and aligns with the OECD System of Health Accounts (SHA 2011) framework. The unit costs represent mean national expenditures per visit and provide standardized population-level estimates of healthcare costs. The visits included: 1) municipal healthcare visits (physician, nurse, physiotherapist, and psychologist); 2) occupational healthcare visits (same providers); and 3) private healthcare visits (physician and physiotherapist).

### Covariates

Covariates included demographic, occupational, and health-related factors collected through self-administered questionnaires during the 46-yr follow-up point of the NFBC1966. Demographic covariates included sex (male and female) and education, categorized as low education (no vocational training, professional course, or vocational school) or high education (college-level training, University of Applied Sciences degree, or university degree). Occupational category (classified into four physical-demand–based groups) and shift work, which was categorized as day work, evening work, two-shift work, or three-shift work.

Body mass index (BMI) was derived from self-reported weight and height by dividing weight in kilograms by the square of height in meters, and then categorized according to WHO criteria (underweight <18.5 kg·m^−2^, normal weight 18.5–24.9 kg·m^−2^, overweight 25–29.9 kg·m^−2^, and obese ≥30 kg·m^−2^) (41). Categorizing BMI is commonly recommended in descriptive analyses and generalized linear models (GLMs) as it improves interpretability (42).

In the final GLM models, only BMI (based on WHO classification) was retained as a covariate. Chronic disease-related indicators (e.g., coronary artery disease, musculoskeletal pain interference, and sickness absence days) were available but were not included as adjustment variables because they are conceptually considered to lie on the causal pathway between sitting and healthcare costs, meaning that adjusting for them would risk overadjustment (16).

### Statistical Analysis

Descriptive statistics were used to summarize sample characteristics: continuous variables are presented as mean ± standard deviation (text) and mean (95% confidence interval [CI]) (tables), and categorical variables as frequencies and percentages. Different categorization strategies were used for descriptive and regression analyses due to their distinct purposes. For descriptive comparisons, sitting time was grouped into three meaningful categories (<4, 4–8, >8 h·d^−1^) to enhance interpretability. For regression models, sitting exposures were divided into quartiles based on their distribution to improve model stability, account for potential nonlinear relationships, and avoid arbitrary cutoffs.

To examine whether domain-specific sitting differed by occupational category, we performed a one-way ANOVA to test the hypothesis that sitting time (work-related, leisure-time, and total) varies across occupational groups. We expected work-related sitting to be highest in office-based occupations and lowest in strenuous labor.

Independent-samples *t* tests were used for binary variables (e.g., sex), and one-way ANOVA for categorical variables with more than two levels (e.g., BMI, occupation, and duration of sitting time categories: low, moderate, and high) when comparing mean sitting time and annual healthcare costs across participant subgroups. These descriptive analyses provided context for interpreting the subsequent GLM analyses.

Annual primary healthcare costs were right-skewed. We therefore analyzed costs using a GLM with a gamma distribution and log link, which is appropriate for modeling positively valued, right-skewed continuous outcomes (43). Because gamma regression requires strictly positive outcome values, zero annual primary healthcare costs (13% of participants) were recoded to €1 to allow inclusion of all participants in the analysis. This pragmatic approach has been used in previous healthcare cost analyses to retain individuals with zero costs, which may carry important information on cost distributions across subgroups (44). Participants with missing covariate data were excluded, resulting in a final analytic sample of 2608 participants for multivariable analysis.

### Additional Analyses

To improve the performance and flexibility of the model, variables (work-related and leisure sitting time, and BMI) were divided into quartiles. Work-related and leisure-time sitting were divided into four categories (quartiles) based on their distribution in the study sample.

Quartile 1 (Q1) represents the group with the lowest sitting time, and Quartile 4 (Q4) represents the group with the highest sitting time. Specifically, work-related sitting was categorized as ≤1.00 h·d^−1^ (Q1), 1.01–3.00 h·d^−1^ (Q2), 3.01–6.50 h·d^−1^ (Q3), and ≥6.51 h·d^−1^ (Q4). Leisure-time sitting was categorized as ≤2.50 h·d^−1^ (Q1), 2.51–3.50 h·d^−1^ (Q2), 3.51–5.00 h·d^−1^ (Q3), and ≥5.01 h·d^−1^ (Q4). The lowest quartile (Q1) was used as the reference category in regression analyses.

Associations between sitting time and annual primary healthcare costs were examined using GLMs. Model 1 included work-related and leisure-time sitting quartiles only. In Model 2, potential covariates (education, self-rated health, and BMI categories) were evaluated for inclusion. These variables were entered additively to control for their influence on the outcome. Based on model performance, only BMI (based on WHO classification; normal weight as reference) was retained in the final adjusted models. Variables that did not improve model performance were not retained.

Results are presented as estimated marginal means (EMMs) with 95% CIs derived from the fitted GLMs. EMMs represent expected healthcare costs for each sitting time quartile while holding covariates constant.

A *P* value < 0.05 was considered statistically significant. All analyses were conducted using IBM SPSS Statistics, Version 27.0 (IBM Corp., Armonk, NY).

## RESULTS

Of the 7147 participants who completed the follow-up questionnaire at the age of 46 yr, 5513 (77.1%) provided valid self-reported data on sitting time. Mean total sitting time was 7.31 ± 3.25 h·d^−1^. On average, participants spent 4.02 ± 2.19 h·d^−1^ sitting during leisure time and 3.40 ± 2.56 h·d^−1^ sitting at work. Within leisure-time sitting, the most common activity was watching television or video content (1.51 ± 1.13 h·d^−1^), followed by sitting in a vehicle (1.03 ± 1.28 h·d^−1^) and using a computer at home (0.95 ± 0.96 h·d^−1^).

Significant differences were observed across education levels (all *P* < 0.001), participants with higher education levels reported more total and work-related sitting time, while those with lower education levels reported more leisure-time sitting. No statistically significant differences were found among BMI or work shift categories. Descriptively, overweight and obese participants had higher average total sitting and leisure-time sitting compared with those of normal weight. No significant differences were found across work shift categories in any sitting domain (Table 1).

**TABLE 1.** The characteristics of the study participants in relation to context-specific and total sitting time (h·d^−1^) at the age of 46 yr

Characteristic	*n* (%)	Work-Related Sitting (Mean, 95% CI)	Leisure Time Sitting (Mean, 95% CI)	Total Sitting Time (Mean, 95% CI)
Education (*n* = 5381)				
No vocational	203 (3.8)	2.10 (1.75–2.45)	5.01 (4.66–5.36)	6.58 (6.09–7.07)
Professional course	242 (4.5)	2.02 (1.72–2.32)	5.07 (4.66–5.48)	6.59 (6.08–7.10)
Vocational school	1816 (33.7)	2.20 (2.09–2.31)	4.25 (4.14–4.36)	6.34 (6.19–6.49)
College-level training	1713 (31.8)	4.16 (4.04–4.28)	3.46 (3.36–3.56)	7.55 (7.40–7.70)
UAS degree	498 (9.3)	4.42 (4.21–4.63)	3.46 (3.33–3.59)	8.19 (7.96–8.42)
University degree	909 (16.9)	5.05 (4.90–5.20)	3.23 (3.14–3.32)	8.25 (8.09–8.41)
AOVA (*F* [df_1_, df_2_], *P*)		*F* (5, 5074) = 156.09, *P* < 0.001	*F* (5, 5325) = 41.49, *P* < 0.001	*F* (5, 5344) = 46.16, *P* < 0.001
BMI (*n* = 4518)				
Underweight	20 (0.4)	3.48 (2.12–4.84)	3.31 (2.69–3.93)	7.07 (5.74–8.40)
Normal weight	1747 (38.7)	3.40 (3.28–3.52)	3.59 (3.49–3.69)	7.26 (7.11–7.40)
Overweight	1980 (43.8)	3.38 (3.27–3.49)	4.03 (3.93–4.13)	7.31 (7.16–7.46)
Obese	771 (17.1)	3.40 (3.19–3.61)	4.02 (3.86–4.18)	7.29 (7.06–7.52)
ANOVA (*F* [df_1_, df_2_], *P*)		*F* (3, 4248) = 0.10, *P* = 0.963	*F* (3, 4467) = 0.67, *P* = 0.572	*F* (3, 4480) = 0.20, *P* = 0.894
Working shifts (*n* = 4686)				
Day job	3728 (79.6)	3.39 (3.31–3.47)	4.02 (3.95–4.09)	7.31 (7.21–7.42)
Evening job	48 (1.0)	4.19 (3.48–4.90)	3.30 (2.93–3.67)	7.33 (6.63–8.03)
Working in two shifts^a^	546 (11.7)	3.3 9 (3.17–3.61)	4.05 (3.86–4.24)	7.35 (7.09–7.61)
Working in three shifts^b^	364 (7.8)	3.41 (3.15–3.67)	4.01 (3.80–4.22)	7.32 (7.00–7.64)
ANOVA (*F* [df_1_, df_2_], *P*)		*F* (3, 4428) = 0.76, *P* = 0.520	*F* (3, 4636) = 0.97, *P* = 0.405	*F* (3, 4649) = 0.06, *P* = 0.981

Values are mean (95% CI).

a Working in two shifts, either morning or evening.

b Working in shifts that include night work (between 23:00 and 06:00 for at least 3 h).

UAS, University of Applied Sciences;

A significant relationship was observed between occupational category and leisure-time sitting. Participants with strenuous work (such as farmers and forest workers) exhibited significantly more leisure-time sitting than office-based workers did, with a mean difference of 1.0 h·d^−1^. Occupational category was significantly associated with work-related sitting time. The highest means were observed among workers whose jobs involved both sitting and walking, followed by office-based workers (Table 2). In line, participants with occupations that involved walking and lifting, and strenuous work reported low work-related sitting time (<4 h·d^−1^).

**TABLE 2.** Mean (95% CI) of total, leisure time, and work-related sitting time hours per day by occupational group at age 46 yr

Occupational Categories	Total Sitting Time (h·d^−1^)	Leisure-Time Sitting (h·d^−1^)	Work-Related Sitting (h·d^−1^)
Office-based workers	7.34 (7.21–7.47)	3.5 (3.42–3.58)	3.6 (3.50–3.70)
Work involving sitting and walking	7.49 (7.11–7.87)	3.5 (3.31–3.69)	4.0 (3.70–4.30)
Work involving walking and lifting	7.26 (7.08–7.44)	4.0 (3.86–4.14)	3.4 (3.26–3.54)
Strenuous work	7.28 (7.04–7.52)	4.5 (4.26–4.74)	2.5 (2.32–2.68)
*F* (df_1_, df_2_) *P* value	*F* (3, 4330) = 1.47 *P* = 0.225	*F* (3, 4330) = 3.15 *P* = 0.031	*F* (3, 4330) = 2.41 *P* = 0.050

### Sitting Time and Primary Healthcare Costs

As shown in Table 3, out of the 4334 participants whose total primary healthcare costs were included in the analysis, 13% (*n* = 562) incurred no costs during a 1-yr period. Across all participants (including those who incurred no costs), the average annual primary healthcare cost was €355 ± 579. Of those who had costs related to healthcare use, the majority (*n* = 1618) reported more than 8 h·d^−1^ of sitting time.

**TABLE 3.** Individual-level self-reported direct healthcare costs by time spent sitting, occupations, gender, and body mass index (BMI). Category healthcare costs (euros, reference year 2011)

Category	Total Cost (Mean €·yr^−1^; 95% CI)	No Costs Incurred, *n*, (%)	Incurred Costs, *n*, (%)
Total sitting time:			
Low (<4 h·d^−1^)	344 (308–380)	95 (11.3)	747 (88.7)
Moderate (4–8 h·d^−1^)	372 (340–404)	211 (13.3)	1379 (86.7)
High (>8 h·d^−1^)	343 (319–367)	253 (13.5)	1618 (86.5)
Occupations:			
Office-based workers	358 (332–384)	241 (13.0)	1619 (87.0)
Work involves sitting and walking	388 (282–494)	24 (11.8)	179 (88.2)
Work involves walking and lifting	354 (320–388)	122 (12.2)	875 (87.8)
Strenuous work	353 (299–407)	72 (13.1)	477 (86.9)
Gender:			
Male	351 (326–376)	263 (12.7)	1805 (87.3)
Female	358 (335–381)	299 (13.2)	1967 (86.6)
BMI:			
Underweight	413 (244–582)	3 (15)	17 (85)
Normal weight	342 (319–365)	216 (12.9)	1462 (87.1)
Overweight	356 (329–383)	252 (13.3)	1641 (86.7)
Obese	376 (325–427)	91 (12.4)	645 (87.6)
Total sample	355 (338–372)	562 (13.0)	3772 (87.0)

Total Costs: Total costs (Mean ± standard deviation) calculated for the sample. No Costs Incurred: Number and percentage of participants with zero healthcare costs. Incurred costs: Number and percentage of participants who reported any healthcare costs during the 1-yr period.

A statistically significant difference was observed in annual total primary healthcare costs across sitting time categories in descriptive analyses (*P* < 0.001), with the highest mean costs observed among participants reporting 4–8 h·d^−1^ of sitting (€372), compared with those with <4 h·d^−1^ (€344) or >8 h·d^−1^ (€343).

According to Table 3, occupations that involved walking and lifting, and strenuous work had the lowest average annual primary healthcare costs: €354 (95% CI = €320–388) and €353 (95% CI = €299–407), respectively. Office-based workers and those working in occupations that required sitting and walking had the highest mean total annual primary healthcare costs: €358 (95% CI = €332–384) and €388 (95% CI = €282–494), respectively. However, the association between occupational group and total costs was not statistically significant. Additionally, average primary healthcare costs varied by BMI category, with the highest costs observed among participants classified as obese or underweight (Table 3).

Associations between sitting time and annual primary healthcare costs were examined in an unadjusted model (Model 1; see Supplemental Tables 1 and 2, Supplemental Digital Content, https://links.lww.com/MSS/D392) and in an adjusted model (Model 2, adjusted for BMI only). Based on model performance, Model 2 was selected for inference.

The GLM with a gamma distribution and log link (Table 4) showed that participants in the second quartile (1.01–3.00 h·d^−1^) of work-related sitting had higher annual primary healthcare costs compared with participants in the lowest quartile (Q1; ≤1.00 h·d^−1^) (*P* = 0.025). EMMs presented in Table 4 indicated adjusted costs of €448 (95% CI: €353–568) in the second quartile, corresponding to an approximately 20% higher cost compared with the lowest quartile of work-related sitting. In addition, participants classified as obese had higher annual primary healthcare costs than participants with normal weight (approximately 20% difference; *P* = 0.026).

**TABLE 4.** Generalized linear Model II (gamma distribution with log link) for annual primary healthcare costs (€), including estimated marginal means (95% CI), reference year 2011

Variables	β (log scale)	SE	*P* Value	Adjusted Mean (€)	95% CI
Work-related sitting categories					
Q1 (ref)	–	–	–	372.98	300.08–462.38
Q2	0.018	0.082	0.025*	448.10	353.25–568.42
Q3	−0.061	0.071	0.397	351.01	280.86–438.68
Q4	−0.035	0.076	0.645	360.14	285.66–454.03
Leisure-time sitting categories					
Q1 (ref)	–	–	–	370.44	295.54–464.32
Q2	0.046	0.076	0.547	387.79	311.09–483.40
Q3	0.007	0.075	0.928	372.97	298.23–466.44
Q4	0.063	0.084	0.458	394.33	311.46–499.25
BMI categories					
Underweight	0.426	0.406	0.295	487.68	220.40–1079.10
Normal (ref)	–	–	–	318.60	290.74–349.14
Overweight	0.114	0.060	0.060	357.15	328.19–388.65
Obese	0.178	0.080	0.026*	380.72	333.46–434.68

Model fit: Deviance/df = 2.48; Pearson χ^2^/df = 2.64; Likelihood Ratio χ^2^ (10) = 19.60, *P* <.001; *N* = 2608.

β, regression coefficient on the log scale; SE, standard error.

Adjusted means (€) represent estimated marginal means derived from generalized linear models with a gamma distribution and log link. Sitting time variables were divided into quartiles (Q1–Q4), with Q1 (lowest sitting time) as the reference group. BMI was categorized according to WHO classification, with normal weight as the reference category.

* *P* < 0.05.

## DISCUSSION

The study examined domain-specific sitting time, specifically sitting during work and leisure, and its associations with annual primary healthcare costs at midlife in a large population-based Finnish cohort. We also explored whether these associations varied by total sitting duration and occupational group. Our findings showed that work-related sitting time in the second quartile (1.01–3.00 h·d^−1^) was associated with higher annual primary healthcare costs compared with the lowest quartile, whereas leisure-time sitting was not independently associated with primary healthcare costs after adjusting for BMI. Participants classified as obese were associated with higher primary healthcare costs. While the average time spent sitting in our sample (7.3 h·d^−1^) aligns with global estimates (ranging from 2.6 to 7.3 h·d^−1^) (45), the observed association between work-related sitting and primary healthcare costs suggests that sitting patterns in different domains may relate differently to healthcare use. Importantly, these findings suggest that the economic implications of SB depend on where sitting occurs, underscoring the value of distinguishing work-related from leisure-time sitting rather than relying on total sedentary time alone.

Behaviorally, prolonged sitting at work often coincides with lower overall physical activity levels, patterns that have been linked to higher healthcare utilization and a greater risk of chronic disease (46). Work environments that limit movement, such as fixed desk-based roles, may contribute to musculoskeletal strain due to sustained static postures, limited mobility, and poor ergonomics (47), while individuals with early functional limitations or emerging health problems may naturally spend more time sitting during work (48). In contrast, leisure-time sitting may reflect recovery from physically demanding work or personal habits that do not consistently increase healthcare costs (49). These behavioral differences may help explain why only a moderate level of work-related sitting in the second quartile was associated with healthcare costs in our study. The absence of associations in the higher quartiles (Q3) may indicate that the relationship between work-related sitting and healthcare use is not linear, with the association appearing only at moderate sitting levels. This interpretation is consistent with previous studies reporting nonlinear or inconsistent associations between work sitting and health outcomes (50)

Physiological mechanisms can also help explain our findings; reduced muscle activity and slower metabolic processes during extended periods of sitting can adversely affect cardiometabolic and musculoskeletal health over time (51). These factors may increase the likelihood that work-related sitting, especially among individuals experiencing early discomfort or emerging health concerns, is more likely to be associated with primary healthcare use.

Our findings extend existing evidence by demonstrating that associations between SB and healthcare costs are domain-specific, highlighting that work-related sitting rather than leisure-time sitting may be a more relevant target for reducing primary healthcare costs in midlife. To our knowledge, this is the first study to examine this relationship in a large, population-based cohort. Previous research highlights leisure-time as a particularly harmful form of SB (52–54). In contrast, our findings did not reveal an independent association between leisure-time sitting and primary healthcare costs after adjusting for BMI. This pattern suggests that the health effects of sitting may depend more on occupational context than on leisure-time sitting alone.

On the other hand, work-related sitting is typically embedded within job roles—particularly desk-based occupations—where long periods of inactivity are difficult to avoid (55,56). Environmental and structural factors, such as limited opportunities to take breaks or move around at work, may further reinforce this pattern. This distinction has practical implications, as reducing work-related sitting may require structural or organizational interventions at the workplace level, whereas leisure-time sitting may be more amenable to individual behavior change. By emphasizing the cost implications specifically linked to work-related sitting, our findings identify potential areas for future workplace and behavioral research aimed at understanding how sedentary time in different domains relates to healthcare use.

We also observed that participants in physically demanding occupations reported lower work-related sitting time but higher leisure-time sitting, which is consistent with prior research (57). This finding suggests that while these workers are less sedentary during work, they may compensate by being more sedentary in their leisure time, potentially due to occupational fatigue. There is also the possibility of reverse association, where healthier individuals select physically demanding jobs, while those with poorer health tend to work in more sedentary job roles.

The groups engaged in strenuous activities have less work-based sitting time, and they constitute a group that may warrant further investigation in studies examining how occupational fatigue influences leisure-time SB. On the other hand, a study of middle-aged Australian women provided contrasting findings, as prolonged sitting time alone showed no significant association with cost after adjusting for other factors (16).

Our study additionally provides new insights, showing that individuals sitting for 4–8 h had the highest average total healthcare costs. Particularly, office-based workers and workers engaged in occupations that mostly involve sitting and walking reported the highest mean total healthcare costs. This aligns with a study that reported significant associations between SB and healthcare costs (14). These observations point to the potential economic burden of SB and highlight the need for further research to understand workplace factors linked to prolonged sitting. Likewise, office-based workers spending relatively more time sitting compared with other occupational categories has several implications. First, the high levels of sitting observed among office-based workers suggest that workplace factors influencing sedentary patterns warrant further investigation. Secondly, there are various work pathways that could explain the observation. Many office-based workers experience prolonged periods of sitting in front of a computer due to the nature of their work. This observation has implications for understanding how occupational environments influence SB and helps identify groups that may need closer examination in future research on the determinants and health impacts of prolonged sitting. These findings are consistent with a previous study, which also reported higher sitting time among office workers (58). This underscores the importance of examining how interruptions during prolonged sitting are related to health outcomes in future research (59). This distinction highlights the importance of considering the context of sitting, such as at work versus during leisure, as its health impacts may vary depending on the domain.

Because BMI was included as a covariate in the adjusted models, we briefly describe its associations with healthcare costs to help interpret the fully adjusted results. We found that the participants classified as obese had higher primary healthcare costs, which is consistent with previous literature showing that BMI is a strong determinant of healthcare utilization and access (60).

### Limitations

Several limitations of this study should be noted. First, sitting time was self-reported, which may introduce recall bias, especially when estimating the time spent in specific domains such as work or leisure. While self-reporting remains a common method in population-based research, it lacks the precision of device-based measurements. Second, sitting time was assessed only once, at the 46-yr follow-up point of the NFBC1966 study, which limits our ability to evaluate longitudinal patterns or causal associations because SB varies over time. Repeated measures would strengthen future analyses. Third, our data capture sitting time only on weekdays, which may underestimate total sedentary time by excluding weekend behaviors, particularly relevant for leisure-time sitting. In addition, domain-specific classifications of sitting time were based on the context of data collection in 2012 and may be era-specific; therefore, generalizability to more recent cohorts with higher prevalence of remote or hybrid work may be limited.

Although data on chronic disease variables were available, they were not included in the final model because they are likely part of the causal pathway between sitting and healthcare costs. As a result, residual confounding by specific health conditions cannot be ruled out, particularly for conditions that may precede higher SB and influence healthcare costs. Additionally, individual income data were not available for the present study at age 46, which limits our ability to fully account for socioeconomic differences in healthcare use. In addition, zero healthcare cost values were adjusted to €1 to enable log-transformation. Although the choice of €1 is somewhat arbitrary, it represents a minimal positive value to retain observations and may influence model estimates and model fit.

In the future, we would also like to include specialized healthcare costs in the analysis. Given the large epidemiological scale of the study, the findings may not capture more granular, context-specific patterns of sitting behavior or individual-level variations and therefore should be interpreted with caution.

On the other hand, this study has several notable strengths. It uses data from a large sample, which enhances the generalizability of the findings to middle-aged adults. The large sample size supports reliable analysis and strengthens the conclusions. A key advantage is the use of domain-specific measures of sitting time, allowing separate examinations of work-related and leisure-time sitting in relation to primary healthcare costs. This approach provides a more detailed understanding of how various types of sitting relate to the occupational context and to the economic burden. The availability of detailed information on participants’ occupations, BMI, and health also enabled adjustment for important confounders in the analysis. Furthermore, the use of a GLM with a gamma distribution and log link appropriately addressed the right-skewed nature of cost data and demonstrated good model fit (deviance/df ≈ 1.08).

## CONCLUSIONS

Higher levels of work-related sitting were associated with higher annual primary healthcare costs, while leisure-time sitting showed no independent association. These findings suggest that the context in which sitting occurs might affect healthcare use. Targeting occupational sitting could be important for future strategies aiming to reduce healthcare costs, but longitudinal studies are needed to determine whether modifying work-related sitting influences healthcare expenditure.

The NFBC1966 46-yr follow-up study received financial support from the University of Oulu (Grant no. 24000692), Oulu University Hospital (Grant no. 24301140), and the ERDF European Regional Development Fund (Grant no. 539/2010 A31592). This work was supported by a scholarship from the Finnish Work Environment Fund.

The authors wish to thank all cohort members, researchers, and NFBC project center personnel who participated in the NFBC data collections. No conflicts of interest were disclosed. The results of the study are presented clearly, honestly, and without fabrication, falsification, or inappropriate data manipulation. The results of the present study do not constitute endorsement by the American College of Sports Medicine.

## Supplementary Material
