# Supplementary material for: Association of Context-Specific Sitting Time with Healthcare Costs at Midlife: The Northern Finland Birth Cohort 1966 Study
Source: Med Sci Sports Exerc. 2026 Mar 26;58(8):1719–28. doi: 10.1249/MSS.0000000000003995 (PMC13331267; doi:10.1249/MSS.0000000000003995)
Supplement: Supplementary file 1 [file msse-58-1719-s001.pdf]

Supplemental Table 1. Generalized linear model I (gamma distribution with log link) predicting annual primary healthcare costs (€), reference year 2011

**Variable**	**Estimate**	**Std. Error**	**Wald X2**	**95 % CI for Wald X2**	**p-value**
**Work-related sitting categories**					
Q2	0.178	0.069	7.170	0.050-0.323	*0.07**
Q3	-0.034	0.060	0.310	-0.152-0.085	0.587
Q4	-0.041	0.065	0.402	-0.169-0.086	0.526
**Leisure-time sitting categories**					
Q2	0.001	0.064	0.00	-0.127-0.125	0.987
Q3	0.031	0.063	0.235	-0.094-0.156	0.628
Q4	0.029	0.070	0.172	-0.109-0.168	0.678

Model fit: Deviance/df = 3.84; Pearson χ^2/df = 3.61; Likelihood Ratio χ^2 (10) = 12.93, $p < .001$; N = 5230. Notes. B = log-scale coefficient; Std.Error = standard error; CI = Confidence Interval. Sitting time variables were divided into quartiles (Q1–Q4), with Q1 (lowest sitting time) used as the reference group. *Values indicate $p < .05$.

Supplemental Table 2. Estimated marginal means with 95% confidence intervals (CIs)

	Model I		
**Variable**	**Mean**	**SE**	**95 % CI**
**Work-related sitting categories**			
Q1	272.14	11.09	251.24–294.77
Q2	328.01	18.52	293.64–366.40
Q3	263.11	11.79	241.01–287.24
Q4	261.13	13.24	236.60–288.21
**Leisure-time sitting categories**			
Q1	275.73	12.69	251.93–301.78
Q2	275.44	12.55	251.91–301.17
Q3	284.39	12.69	260.57–310.39
Q4	283.95	12.69	251.93–301.78

Values are estimated marginal means (95% CI) derived from generalized linear models with a gamma distribution and log link. Model 1 includes main exposure variables only. Sitting time variables were divided into quartiles (Q1–Q4), with Q1 (lowest sitting time) used as the reference category. SE = standard error.
